# TRPV1 deletion impaired fracture healing and inhibited osteoclast and osteoblast differentiation

**DOI:** 10.1038/srep42385

**Published:** 2017-02-22

**Authors:** Lin-Hai He, Meng Liu, Yang He, E. Xiao, Lu Zhao, Ting Zhang, Hua-Qian Yang, Yi Zhang

**Affiliations:** 1 Department of Oral and Maxillofacial Surgery, Peking University School and Hospital of Stomatology; National Engineering Laboratory for Digital and Material Technology of Stomatology, Beijing Key Laboratory of digital Stomatology, Beijing, China; 2 Center for Craniofacial Stem Cell Research and Regeneration, Department of Orthodontics, Peking University School and Hospital of Stomatology; National Engineering Laboratory for Digital and Material Technology of Stomatology, Beijing Key Laboratory of digital Stomatology, Beijing, China; 3 State Key Laboratory of Biomembrane and Membrane Biotechnology, College of Life Sciences, Peking University, Beijing, China

## Abstract

Fracture healing, in which osteoclasts and osteoblasts play important roles, has drawn much clinical attention. Osteoclast deficiency or decreased osteoblast activity will impair fracture healing. TRPV1 is a member of the Ca^2+^ permeable cation channel subfamily, and pharmacological inhibition of TRPV1 prevents ovariectomy-induced bone loss, which makes TRPV1 a potential target for osteoporosis. However, whether long term TRPV1 inhibition or TRPV1 deletion will affect the fracture healing process is unclear. In this study, we found that the wild-type mice showed a well-remodeled fracture callus, whereas TRPV1 knockout mice still had an obvious fracture gap with unresorbed soft-callus 4 weeks post-fracture. The number of osteoclasts was reduced in the TRPV1 knockout fracture callus, and osteoclast formation and resorption activity were also impaired *in vitro*. TRPV1 deletion decreased the calcium oscillation frequency and peak cytoplasmic concentration in osteoclast precursors, subsequently reducing the expression and nuclear translocation of NFATc1 and downregulating DC-stamp, cathepsin K, and ATP6V. In addition, TRPV1 deletion caused reduced mRNA and protein expression of Runx2 and ALP in bone marrow stromal cells (BMSCs) and reduced calcium deposition *in vitro*. Our results suggest that TRPV1 deletion impairs fracture healing, and inhibited osteoclastogenesis and osteogenesis.

Bone is a rigid yet dynamic organ that is continuously remodeling, and osteoblasts and osteoclasts exert indispensable functions^1^. Many skeletal diseases, such as osteoporosis, are a result of excess osteoclast activity or decreased osteoblast activity^2^. Fracture healing is a complex and dynamic process being governed by a variety of cellular elements and cytokines^3^,^4^, in which osteoclasts and osteoblasts are the two important cell types^3^. Osteoclasts were responsible for cartilage resorption and remodeling during fracture healing by secreting acid and proteinases while osteoblasts form new bone^5^. Enhancing osteoclastogenesis would accelerate cartilage resorption, and increasing osteoblastogenesis during fracture healing would promote bone union, whereas inhibition of osteoclast or osteoblast differentiation are reported to delay bone healing^6^,^7^. Osteoclastogenesis and osteoblastogenesis are regulated by many factors, of which calcium signaling is an important one^8^. Calcium not only serves as an extracellular messenger that controls osteoclast and osteoblast function but also plays a critical role in cell signaling^9^,^10^. On the one hand, osteoclasts can sense their ambient Ca^2+^ level during resorption and delicately adjust the bone resorption process^11^. On the other hand, RANKL signaling can evoke Ca^2+^ oscillations, resulting in Ca^2+^/calcineurin-dependent nuclear factor-activated T cells c1 (NFATc1) activation and prompt osteoclast differentiation^12^. For osteoblasts, NFATc1, activated by calcium signaling pathways, functions as an important transcription factor to promote osteoblast differentiation^13^,^14^,^15^.

The transient receptor potential vanilloid (TRPV) subfamily is Ca^2+^ permeable cation channel, which can be activated by heat, protons, swelling, and other stimuli^16^. Osteoclasts express TRPV1, TRPV4, TRPV5, and TRPV6^17^,^18^,^19^. The genetic knockout or pharmacological inhibition of TRPV1 was reported to protect against ovariectomy-induced bone loss^20^,^21^, which suggests that TRPV1 is a potential target for preventing osteoporosis. Another study found that TRPV1 was also expressed in osteoblasts and that capsazepine, which is a TRPV1 inhibitor, impaired osteoblast differentiation ability^20^. However, whether long-term TRPV1 inhibition or deletion will affect the fracture healing process is still unknown.

In this study, we aimed to explore the effect of TRPV1 deletion on fracture healing and to elucidate the mechanism of how TRPV1 deletion affects osteoclastogenesis by using TRPV1 knockout mice.

## Results

### TRPV1 gene deletion increased physiological bone density

**Figure 1:**
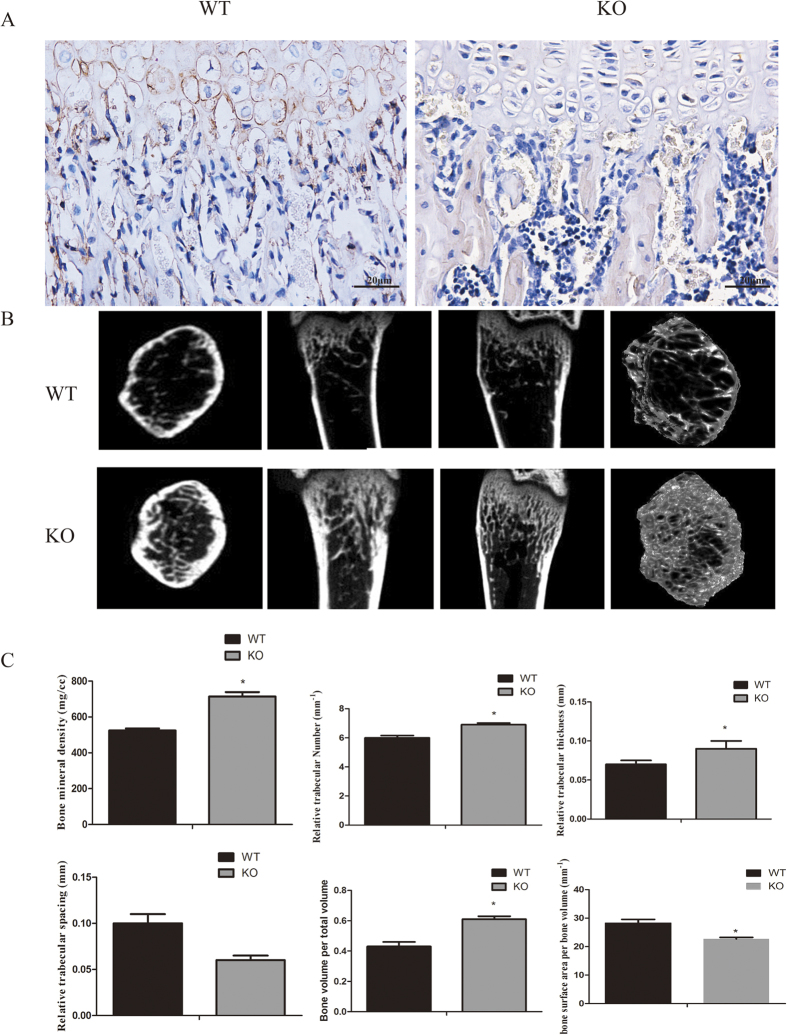
TRPV1 gene deletion increased bone density. (**A**) TRPV1 expression in WT bone marrow and chondrocytes while no TRPV1 expressed in KO group. (**B**) microCT images showed the bone structure of TRPV1 knockout group and WT group. (**C**) the quantitative analysis of microCT results. (*p < 0.05).

To confirm the TRPV1 knockout efficiency, we conducted the immunohistochemistry to detect the TRPV1 expression in bone marrow. The results showed that TRPV1 was expressed in WT bone marrow and chondrocytes but not TRPV1 knockout tissues (Fig. 1A). To assess the effect of TRPV1 deletion on the bone remodeling process, we evaluated bone quality by microCT analysis. The results showed that TRPV1 knockout mice had a high bone density compared with wild-type mice (Fig. 1B). The quantitative results of microCT scans showed that TRPV1 knockout mice had higher bone mineral density (WT vs KO, 524.5 ± 11.14 vs 713.9 ± 25.01, mg/cc), bone volume/total volume (WT vs KO, 0.43 ± 0.03 vs 0.61 ± 0.02), trabecular thickness (WT vs KO, 0.07 ± 0.01 vs 0.11 ± 0.02 mm) and trabecular number (WT vs KO, 6.00 ± 0.16 vs 6.90 ± 0.02, 1/mm) accompanied by lower bone surface area/bone volume (WT vs KO, 28.26 ± 1.28 vs 22.67 ± 0.58, 1/mm) and trabecular spacing (WT vs KO, 0.11 ± 0.02 vs 0.06 ± 0.01 mm) than WT mice (Fig. 1C), which implied that TRPV1 deletion impaired the bone remodeling process.

### TRPV1 deletion increased fracture callus volume and impaired fracture healing

**Figure 2:**
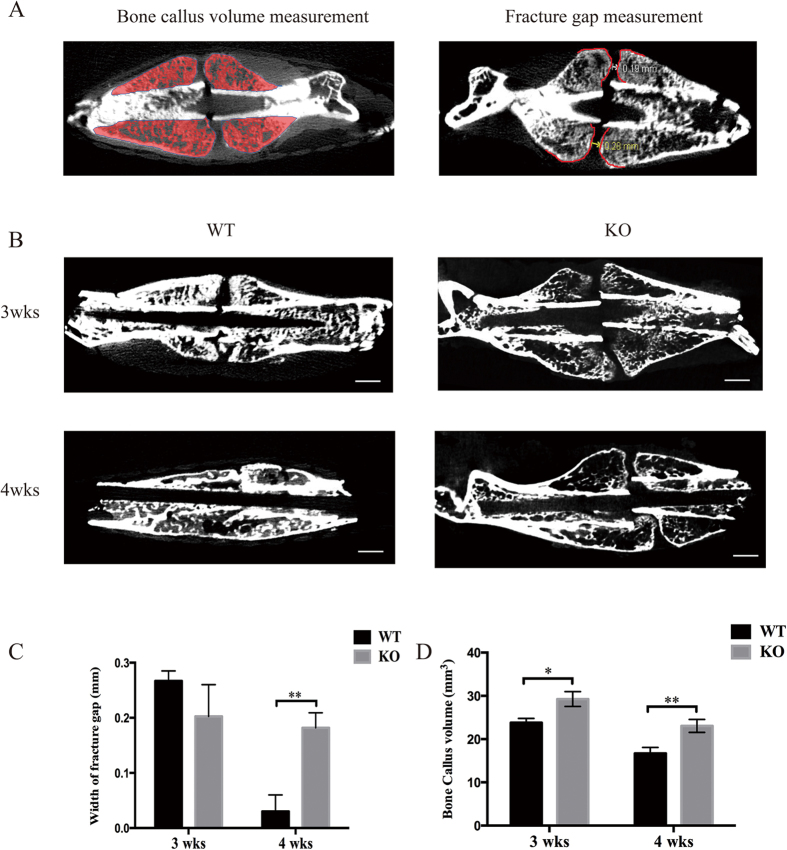
TRPV1 knockout delayed fracture healing in microCT images. (**A**) the left panel shows the ROI and how the fracture callus volume was measured in this study; the right panel showed the ROI and how the fracture gap was measured. (**B**) micro-CT images showed the callus gaps and volumes 3 and 4 weeks post-fracture. (**C**) the left graph shows the quantitative width of fracture gap. The right graph shows the quantification of bone callus volume (WT: wild-type group; KO: knockout group).

The fracture healing process is conventionally partitioned into four stages, and 3 weeks post-fracture and 4 weeks post-fracture are widely used timepoints to assess the fracture healing process^6^. Our results showed that 3 weeks post-fracture, the TRPV1 knockout mice showed larger fracture calluses (29.23 ± 1.73 mm^3^ vs. 23.77 ± 1.00 mm^3^), and both groups showed comparable fracture gaps (Fig. 2B). After 4 weeks post-fracture, all the fracture calluses of the wild-type group showed bony connective junctions between the fracture gaps, and the callus size was smaller, whereas the TRPV1 knockout fracture callus still had an obvious fracture gap (WT vs. KO, 0.03 ± 0.03 mm vs. 0.18 ± 0.03 mm) (Fig. 2B and C) and showed a larger callus (KO vs. WT, 23.01 ± 1.50 mm^3^ vs. 16.70 ± 1.40 mm^3^) (Fig. 2D). The results showed that TRPV1 deletion impaired fracture healing.

### TRPV1 deletion decreased osteoclast number and delayed soft-callus remodeling

**Figure 3:**
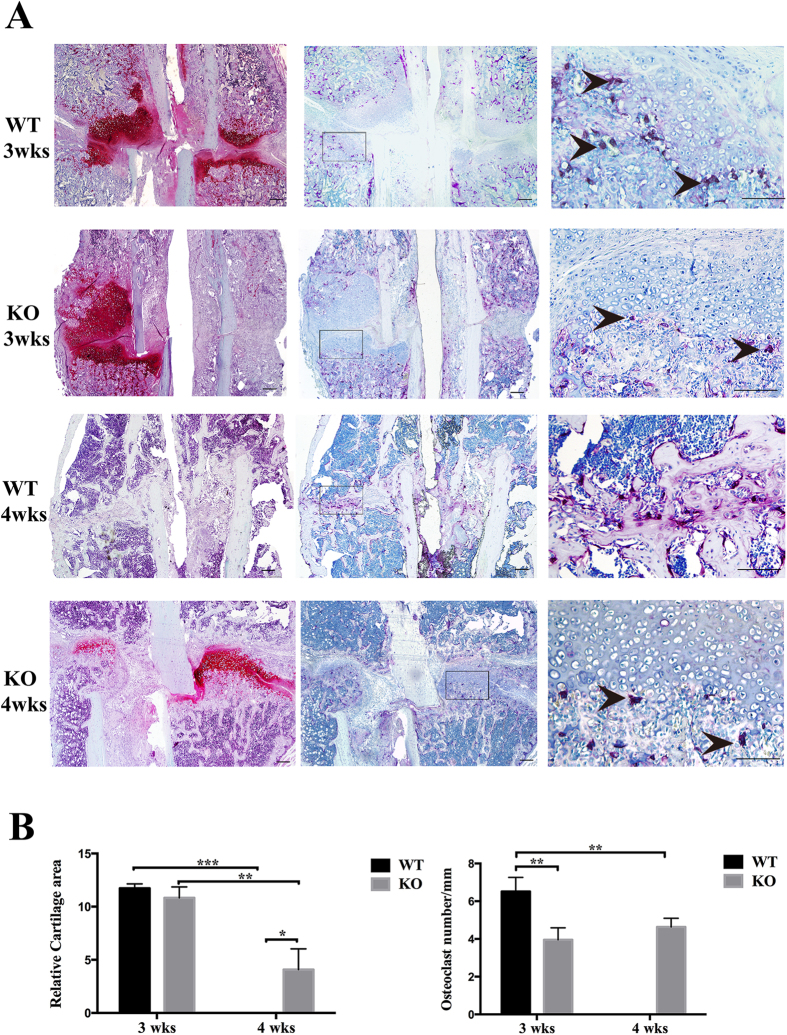
TRPV1 knockout decreased the number of TRAP-positive cells and inhibited cartilage dissolution. (**A**) The left four pictures are safranin O stained and showed the red color cartilage (bar = 200 μm). The middle four (bar = 200 μm) and right four (bar = 100 μm) pictures are TRAP stained. The right four pictures show magnified views of the black frames in the middle pictures. The 4 weeks post-fracture WT group showed that the cartilage had been removed and exhibited hard callus remodeling. The black arrowhead showed TRAP-positive osteoclasts at the cartilage-bone boundary (WT: wild-type group, KO: knockout group). (**B**) The left graph is the quantification of the relative cartilage area. The right graph is the quantification of the number of TRAP-positive cells (*p < 0.05, **p < 0.01, and ***p < 0.001). note: the osteoclasts were quantified near the cartilage-bone boundary and the and the osteoclast in the bone marrow and hard-callus were not include in this study.

To explore the mechanism of delayed fracture healing in the TRPV1 knockout group, we conducted safranin O staining. Figure 3A shows that 3 weeks post-fracture, the wild-type group and TRPV1 knockout group had comparable cartilage callus sizes (WT vs. KO = 11.73 ± 0.42 mm^2^ vs. 10.84 ± 1.02 mm^2^) (Fig. 3A and B), but 4 weeks post-fracture, the cartilage of the wild-type group was entirely replaced by bony callus. However, the TRPV1 knockout group still had cartilaginous calluses (WT vs. KO = 0 ± 0 mm^2^ vs. 4.08 ± 1.94 mm^2^) (Fig. 3B). Considering that osteoclasts exert an important role during soft-callus remodeling, we assessed osteoclast formation near the chondroosseous junctions. The result showed that there were much osteoclasts near cartilage-bone boundary of WT group while less osteoclasts existed near cartilage-boundary of KO group at 3-weeks postfracture. The quantification results showed that TRPV1 knockout group had significantly less TRAP-positive osteoclasts near cartilage-bone boundary compared with wild-type group (KO vs. WT = 3.96 ± 0.32 per mm vs. 6.51 ± 0.75 per mm) (Fig. 3A and B). 4-weeks postfracture, the WT group exhibited hard-callus remodeling and there were much osteoclasts at the hard-callus while KO group still exhibited soft-callus and the less osteoclasts near cartilage-bone boundary Note: note: the osteoclasts were quantified near the cartilage-bone boundary and the and the osteoclast in the bone marrow and hard-callus were not include in this study. These results showed that decreased numbers of osteoclasts are involved in the delayed soft-callus remodeling.

### TRPV1 deficiency impaired osteoclastogenesis *in vivo* and *in vitro*

**Figure 4:**
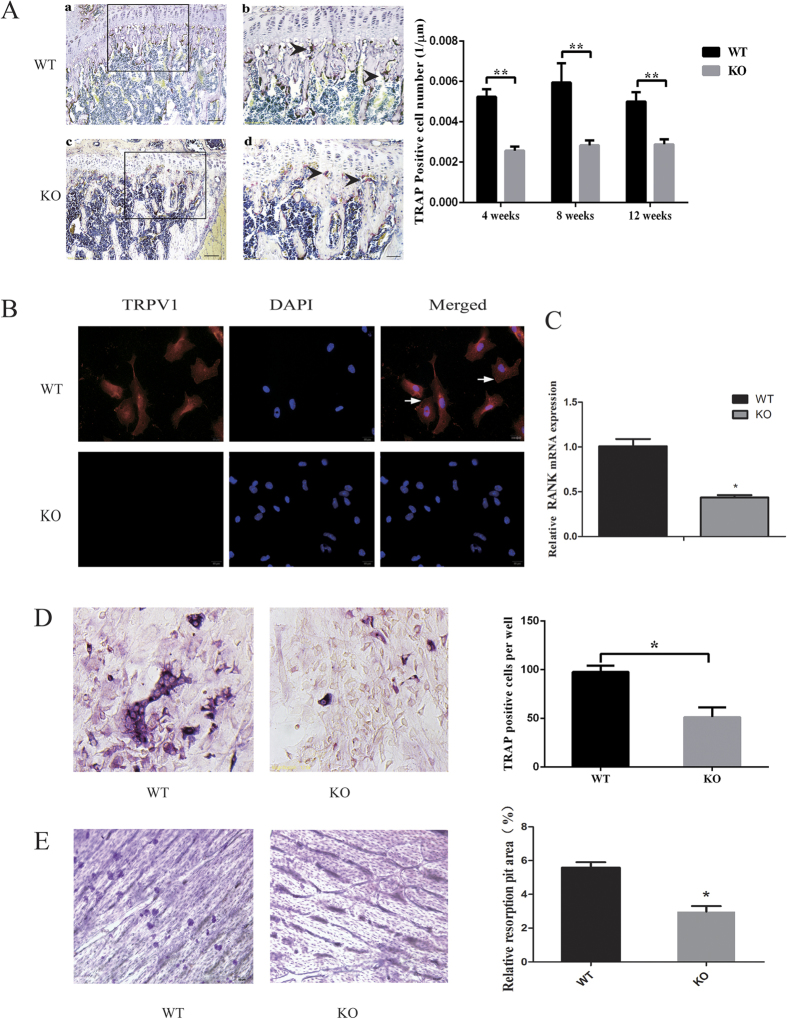
TRPV1 knockout impaired osteoclastogenesis *in vivo* and *in vitro*. (**A**) TRAP staining. The left images show that the knockout group had fewer TRAP-positive cells at 8 weeks compared with the wild-type group. Figure Ab,d show a magnified view of the black frames in Fig. Aa,c, respectively, and the black arrowheads showed the TRAP-positive osteoclasts (bar = 200 μm in Fig. Ca,c; bar = 100 μm in Fig. Ab,d). The right graph shows the quantification of TRAP-positive cells per length. (**B**) The cell immunofluorescence images showed that TRPV1 was expressed in WT osteoclast precursors but not in those from the TRPV1 KO group. (**C**) RANK mRNA expression in the TRPV1 knockout group and WT group by real-time PCR assays. (**D**) The left images show that the TRPV1 knockout group has fewer TRAP-positive cells *in vitro* (bar = 50 μm). The right graph shows the qualification of TRAP-positive cell number after *in vitro* osteoclastogenic induction. (**E**) The left images showed that WT osteoclasts formed more resorption pits on the bone slice than those from the TRPV1 KO group. The right graph shows the quantification of the relative resorption area (*p < 0.05, **p < 0.01).

The above results showed that the number of osteoclasts was reduced during the fracture healing process. In addition, we assessed the osteoclasts in the normal femurs of both groups. Interestingly, the TRAP staining results showed that during bone development, the TRPV1 knockout group also had significantly fewer TRAP-positive osteoclasts *in vivo* not only at the age of 8 weeks (Fig. 4A) (2.83 ± 0.24 per mm vs. 5.94 ± 0.97 per mm) but also at 4 weeks (2.57 ± 0.21 per mm vs. 5.24 ± 0.37 per mm) and 12 weeks (2.89 ± 0.25 per μm vs. 5.00 ± 0.46 per mm). We hypothesized that TRPV1 knockout might directly impair osteoclast differentiation. To test this hypothesis, we isolated the bone marrow monocytes from both groups to eliminate the effect of the bone marrow niche on osteoclastogenesis. With regard to osteoclastogenic induction, TRPV1 was expressed in wild-type osteoclast precursors (Fig. 4B) but not in the knockouts; furthermore, RANK mRNA expression was also decreased in the TRPV1 KO group (Fig. 4C). The results showed that osteoclast precursors derived from TRPV1 knockout mice formed fewer osteoclasts per well under Rankl stimulation, and the osteoclast resorption ability of TRPV1 knockout group was also impaired (Fig. 4D and E). These results suggested that osteoclast differentiation and resorption capacity were impaired in the TRPV1 knockout group.

### TRPV1 deletion downregulated the activation of NFATc1 during osteoclast differentiation

**Figure 5:**
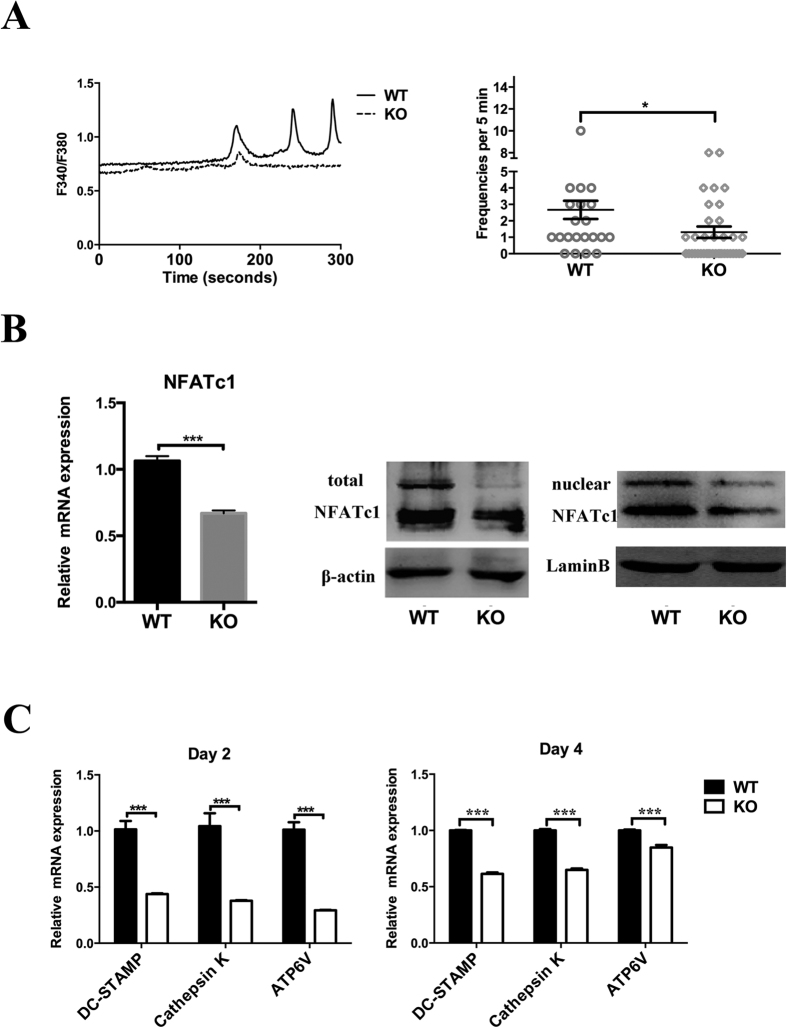
TRPV1 knockout inhibits calcium signal downstream NFATc1. (**A**) The left graph shows the time course of the calcium oscillations of both groups. The right graph shows the summary of all the calculated cells of both groups. (**B**) The left graph shows the real-time PCR results from BMMs induced to osteoclastogenesis for 2 days. The right images show western blots of total and nuclear NFATc1. (**C**) Real-time PCR results show the NFATc1 target genes DC stamp, cathepsin K, and ATP6V mRNA expression (*p < 0.05 and ***p < 0.001; WT: wild-type group, KO: knockout group).

Osteoclast differentiation was impaired *in vitro* and *in vivo*. However, how TRPV1 deficiency affected osteoclastogenesis was not clear. Considering that TRPV1 is a Ca^2+^ -selective cation channel, the calcium oscillation frequency and oscillation peak were important for the calcium signal cascade. Thus, we assessed the basal calcium oscillation frequency and oscillation peak, and the results showed that TRPV1 deficiency resulted in a decreased calcium oscillation peak value and wave number, and the mean calcium oscillation frequency in the TRPV1 deficiency group was also reduced (1.31 ± 0.32 per 5 min vs. 2.67 ± 0.55 per 5 min) in the TRPV1 deficiency group compared with the wild-type osteoclast precursors after Rankl stimulation for 2 days (Fig. 5A). NFATc1 is an important downstream transcription factor in calcium signaling and is essential for osteoclastogenic-related gene expression. We evaluated the total NFATc1 expression by real-time PCR and western blotting. The results showed that the total NFATc1 was significantly decreased both at the mRNA (0.67 ± 0.02 vs. 1.06 ± 0.36) and protein levels. Nuclear NFATc1 also decreased in the TRPV1 knockout group (Fig. 5B). Real-time PCR showed decreased mRNA expression of DC-stamp, cathepsin K, and ATP6V on both days 2 and 4 after osteoclastogenic induction of the bone marrow monocytes (Fig. 5C). These findings showed that TRPV1 deletion impaired NFATc1 signaling.

### TRPV1 KO BMSCs exhibited less osteogenic potential than WT BMSCs

**Figure 6:**
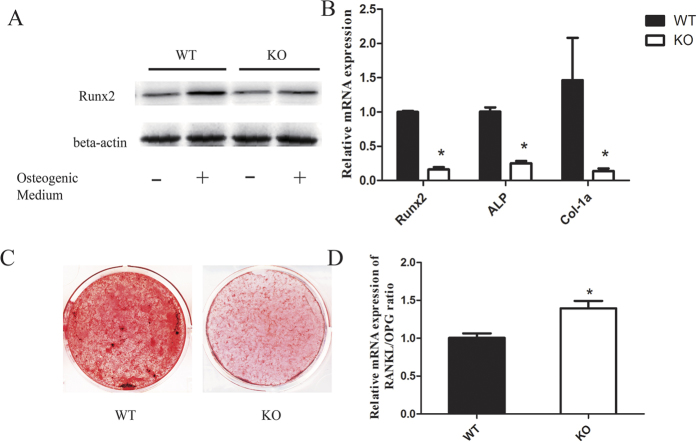
TRPV1 deficiency impaired the osteogenic potential of BMSCs. (**A**) Western blot analysis of Runx2 and ALP expression in BMSCs after osteogenic induction for 7 days. (**B**) osteogenic-related mRNA expression of genes in BMSCs was quantitatively evaluated via real-time PCR. (**C**) alizarin red staining of calcium deposition of BMSCs after osteogenic induction for 21 days. (**D**) relative RANKL/OPG mRNA expression ratio evaluated via real-time PCR analysis.

As the osteogenic potential of BMSCs is another important factor during fracture healing, and calcium signaling is also important for osteoblast differentiation, we wondered whether TRPV1 deletion affected the bone formation ability of BMSCs. The results showed that WT BMSCs expressed higher levels of Runx2 and ALP than TRPV1 KO BMSCs (Fig. 6A and B). Alizarin red staining results showed that the BMSCs from the TRPV1 KO group deposited less calcium than those from the WT group (Fig. 6C). Additionally, the TRPV1 knockout BMSCs showed a slightly increased RANKL/OPG ratio during osteoblast differentiation (Fig. 6D). These results showed that TRPV1 deletion impaired the osteogenic potential of BMSCs.

## Discussion

In this study, we first showed that TRPV1 deletion impaired osteoclastogenesis and osteoblast differentiation, which might contribute to delayed fracture healing.

Osteoclasts are essential for bone homeostasis and fracture healing. Bone fracture healing is fracture healing is a complex and dynamic process being governed by a variety of cellular elements and cytokines^3^,^4^, disorganized cellular or microenvironment will lead to abnormal fracture healing^22^,^23^,^24^. The replacement of a cartilaginous callus by hard callus is the typical endochondral ossification process and in which osteoclasts played an important role^25^,^26^. Cartilage dissolution was disrupted by the inhibition of osteoclast formation and delayed fracture healing, with distinct fracture gaps and more callus cartilage in the soft-callus remodeling stage^27^; the reduction in the number of osteoclasts during fracture healing contributes to a larger fracture callus size^25^,^28^. Osteoclast formation promoted by the knockout of OPG accelerates fracture healing^26^. Our results showed that TRPV1 knockout mice exhibited higher bone density than WT mice with higher bone mineral density, bone volume/total volume, trabecular thickness and trabecular number at physiological status which suggested TRPV1 deletion impaired bone remodeling process. Consistently, Rossi *et al*. and Idris *et al*. reported that TRPV1^−/−^ mice had higher bone density and TRPV1 inhibition prevents ovariectomy-induced bone loss by affecting osteoclast and osteoblast differentiation^20^,^21^.Our results showed that TRPV1 knockout mice exhibited higher bone density than WT mice, with higher bone mineral density, bone volume/total volume, trabecular thickness and trabecular number at a physiological status, which suggested that TRPV1 deletion impaired the bone remodeling process and we found that TRPV1 was increased in fracture healing which suggested TRPV1 was involved in fracture healing process (supplemental Fig. 1). Soft-callus remodeling is also an important process during fracture healing; thus, we supposed that TRPV1 deletion might impair fracture healing. As expected, the results showed that TRPV1 knockout mice exhibited distinct fracture gaps and increased callus size 4 weeks post-fracture. The wild-type group and the TRPV1 knockout group had comparable cartilage 3 weeks post-fracture, but at 4 weeks post-fracture, the cartilage of the wild-type group was mostly resorbed whereas the TRPV1 knockout group still had considerable cartilage, which means that the delayed fracture healing is not due to the initial size of the formed soft-callus but due to the soft-hard callus transformation. Interestingly, TRPV1 positive osteoclasts were increased during fracture healing (supplemental data1) and this implied that osteoclasts exert essential roles during the cartilage removal process. The number of osteoclasts was reduced near the chondroosseous junctions in the TRPV1 knockout group, which implies that reduced number of osteoclasts might be involved in the delayed fracture healing by decreased cartilage resorption. A previous study had found that anti-resorptive therapies can lead to larger callus sizes caused by the delayed resorption of the mineralized cartilage^25^. Thus, impaired osteoclastogenesis during fracture healing might also contribute to larger callus size in the TRPV1 deletion group. Moreover, we assessed the osteoclast formation during bone development and found that osteoclast formation was also decreased. To eliminate the effects of the bone marrow niche on osteoclastogenesis, we conducted *in vitro* assays, and the results suggested that TRPV1 deletion directly inhibited osteoclastogenesis and resorptive capacity. Interestingly, RANK expression was decreased in TRPV1-deleted pre-osteoclasts, which might contribute to reduced osteoclastogenesis. These results were consistent with a previous study^20^,^21^, which suggested that the TRPV1 inhibitor, capsazepine, suppressed osteoclast formation *in vitro*. Though decreased osteoclasogenesis might be a potent mechanism leading to impaired fracture healing in TRPV1 knockout group, other factors, osteoblast, nerve cells, cytokines and so on, might also be involved in the impaired fracture healing process^29^. However, there were no significant changes of TNF-α and IL-1β expression between WT group and TRPV1 group (see in supplemental Fig. 2). We supposed that inflammation factors might not play the key role in TRPV1 deletion induced impaired fracture healing. Although a few studies had suggested that the TRPV subfamily exerts essential roles in maintaining bone architecture, we are the first to report that deletion of TRPV1, a member of the TRP subfamily, affects fracture healing. Our results might help clinicians to assess the application of TRPV1-targeted drugs during fracture healing.

TRP-family channels are calcium-selective cation channels. RANKL signaling can evoke Ca^2+^ oscillations, resulting in Ca^2+^/calcineurin-dependent NFATc1 activation and prompt osteoclast differentiation^12^. Previous studies have shown that the genetic knockout of TRPV5 can inhibit the resorption function of osteoclasts^18^. *In vivo* knockout of the TRPV4 gene could inhibit osteoclast differentiation and increase bone mineral density^17^. Although a previous study suggested that TRPV1 deficiency or inactivation impaired osteoclast activity^20^,^21^, the mechanism of how TRPV1 deficiency inhibits osteoclastogenesis was unclear. Previously, Orita S *et al*. and Yoshino K *et al*. reports that TRPV1 was increased in dorsal root ganglia neurons in ovariectomy-induced osteoporosis^30^,^31^. We showed that the osteoclast precursor cells of the TRPV1 knockout group had reduced calcium oscillations and peak concentrations after stimulation with RANKL for 2 days. However, the TRPV1 knockout group osteoclast precursors still exhibited weak calcium oscillation, which suggested that the calcium oscillation was not completely dependent on TRPV1 and that other TRP channels might be involved in the calcium oscillation of osteoclasts^32^,^33^,^34^. Two calcium-dependent signaling pathways may be related to NFATc1. First, the Ca^2+^/calmodulin pathway induces *c-fos* expression, which increases NFATc1 transcription^35^. Second, the Ca^2+^ -dependent activation of calcineurin leads to NFATc1 nuclear translocation^8^. Thus, we directly assessed NFATc1 expression and nuclear translocation. Interestingly, the results showed that both NFATc1 expression and nuclear translocation were reduced. These results could explain how in our study, impaired calcium signaling significantly reduced not only the total NFATc1 but also the nuclear NFATc1 protein in the TRPV1 knockout group. In another study, knockout of TRPV4, the homolog of TRPV1, also leads to impaired osteoclastogenesis by affecting the downstream calcium signaling transcription factor NFATc1^17^. In addition, the reduced expression of DC-stamp, cathepsin K, and ATP6V, which are all reported to be target genes of NFATc1^36^,^37^,^38^, confirmed the inhibition of NFATc1 in TRPV1 knockout mice. At the same time, we compared the osteoclastogenic potential of BMMs derived from the wild-type group and the TRPV1 knockout group and found that the TRPV1 knockout group BMMs also exhibited impaired osteoclastogenesis. Thus, the knockout of TRPV1 leads to decreased osteoclastogenesis through the down-regulated expression and activation of NFATc1.

TRPV1 is also expressed in bone-forming cells, and bone formation is another important factor during fracture healing. During fracture healing, osteoblasts form new bone and promote osteoclast formation by upregulating Rankl/OPG to remove the cartilage^5^. Thus, we evaluated the effect of TRPV1 knockout on the bone formation capacity of BMSCs. Our results suggested that TRPV1 deficiency also impaired the osteogenic potential of BMSCs, which was consistent with previous studies^39^, and this might be one potential factor leading to delayed fracture healing. However, the mechanism by which TRPV1 deletion impaired osteoblast differentiation requires further research.

In conclusion, TRPV1 deletion impaired fracture healing, and decreased osteoclast and osteoblast differentiation.

## Materials and Methods

### Animals

TRPV1 knockout mice (TRPV1 KO, B6.129 × 1-*TRPV1*^*tm1Jul*^*/*J, 003770) were purchased from the Jackson Laboratory (The Jackson Laboratory, JAX^®^ Mice and Services, Bar Harbor, ME, USA). The genotype was tested by polymerase chain reaction (PCR) following the instructions of the Jackson Laboratory. According to the Jackson Laboratory description, TRPV1 knockout mice are healthy, fertile, and indistinguishable from their age- and sex-matched C57BL-6J wild-type controls in terms of size and weight.

### Ethics statement

The Ethics Committee of the Peking University Health Science Center approved this study (approval number: LA2014228). The mice were anesthetized by intraperitoneal injection of 2% sodium pentobarbital during the fracture surgery or were sacrificed by euthanasia. Food and water were provided to the mice ad libitum during fracture healing. Tramadol hydrochloride was added to the drinking water (25 mg/L) to avoid post-procedural pain. All mice involved in this study were alive and healthy, and the wounds healed well at the experimental endpoint. All experiments were carried out in accordance with Animal Experimentation Ethics Guidelines of the Peking University Health Science Center.

### Animal fracture model

To assess the effect of TRPV1 deletion on the fracture healing process, a unilateral open bone fracture model was adopted in this study. Two groups, a group of eight 6-week-old male WT mice and a group of eight 6-week-old male TRPV1 knockout (TRPV1 KO) mice, were used. Briefly, after anesthesia, the operation area was shaved and disinfected. Then, a 7 to 8 mm longitudinal skin incision from knee to hip was made on the lateral side of the mid-thigh, and the mid-diaphyseal of the left femur was carefully exposed by blunt dissection of the muscles while sparing the sciatic nerve. The femur was stabilized with finely curved forceps. An osteotomy was made in the middle of the femur using a reinforcement fiber glass disc (2000 rpm/min) with normal saline cooling. Afterward, a 25-G spinal needle was inserted into the femur for fixation. After fixation, the wound was closed by layers. After skin closure, the mice were returned to their cages, and postoperative analgesia was conducted. The critical period for transformation from soft callus to hard callus was between 3 and 4 weeks post-fracture^6^,^25^. Thus, the mice were euthanized after 3 and 4 weeks. The femurs were isolated for biomolecular, microCT, and morphological analyses.

### Microscopic computerized tomography (micro-CT) evaluation

TRPV1 knockout and wild-type group mouse at age of 4 weeks were sacrificed. Femurs were fixed in 10% formalin for 24 hours and then scanned with Micro-CT system. TRPV1 knockout and wild-type fracture calluses from 3 or 4 weeks post-fracture were fixed in 10% formalin for 24 h and then scanned with a micro-CT system (Inveon, Simens, Germany) at 60 kV, 220 μA, 500 ms exposure time, and 882 μm effective pixel size. The images were analyzed with Inveon Research Workplace software (Inveon MM, Simens, USA). The bone mineral density, bone volume per total volume, the mean trabecular thickness, the mean trabecular number, the mean trabecular spacing, the bone surface area per bone volume were calculated by this software as previous methods^40^.

#### Callus gap measurement

For the microCT, every tenth slice of fracture callus (50 μm interval) from the first slice to the last slice in the bone axial plane was chosen. The distance was measured three times for each slice between the callus outline midpoints of both callus ends (see Fig. 2A), and the mean value was used for statistical analysis.

#### Callus volume measurement

All the slices used for the microCT images were included in the measurement. In each slice, the fracture callus except the cortex bone was chosen, and all the slices were eventually integrated. The bone parameters were calculated by the Inveon Research Workplace software (Inveon MM, Siemens, USA) (region of interest is shown in Fig. 2A).

### Safranin O staining

Paraffin-embedded, 3 μm thick sections were deparaffinized in xylene, rehydrated in an alcohol gradient, and rinsed in distilled water. Sections were stained with hematoxylin working solution for 5 min, washed in running tap water for 10 min, rinsed quickly with 1% acetic acid solution for 15 s, and stained in 0.1% safranin O solution for 5 min. The sections were dehydrated and cleared with 95% ethyl alcohol, absolute ethyl alcohol, and xylene and mounted using resinous medium.

### *In vivo* and *in vitro* tartrate-resistant acid phosphatase staining

TRPV1 knockout and wild-type group mice were sacrificed by euthanasia at 4, 8, and 12 weeks of age. Femurs were fixed in 10% formalin for 24 h and then decalcified in 10% EDTA for 6 weeks. EDTA was changed twice a week. The tissues were then embedded in paraffin. Three random 3 μm thick sections were cut and used for TRAP staining (Sigma-Aldrich, St. Louis, MO, USA) following the manufacturer’s instructions.

Bone marrow cells were differentiated after 4–5 days of M-CSF and RANKL stimulation, fixed in 10% formalin for 10 min, and then stained with TRAP using the same TRAP kit (Sigma Diagnostics, USA) according to the manufacturer’s instructions. The TRAP^+^ osteoclasts were counted in 5 wells of a 96-well plate, and the data were presented as osteoclast number per well.

### Quantification of cartilage area and osteoclast number

The cartilage area and the relative osteoclast number quantification were measured with a BIOQUANT OSTEO Bone Biology Research System (Bioquant Image Analysis Corporation, USA) according to the manufacturer’s instructions. Briefly, three sections used for safranin O staining and TRAP staining were selected randomly from the middle part of the tissue, and the whole view of the safranin O staining images at the fracture sites was captured using a microscope (Leica Microsystems, Germany). The data were transferred to a Bioquant Osteo Bone Biology Research System. The bone morphology quantification and analysis list was chosen. The orange-colored remnant cartilage between the fracture gaps was selected, and the cartilage area was calculated and analyzed by the software. The relative cartilage area was presented as mm^2^.

Images of the entire TRAP-stained bone–cartilage boundary of the fracture sites were captured, and the relative osteoclast number was measured with the bone cell quantification and analysis system list. Briefly, the chondroosseous junction was depicted, and the rose-colored osteoclasts were located. The relative osteoclast number was calculated as osteoclast number per chondroosseous junction length, and the data were presented as osteoclast number per mm. All the data were replicated twice, and the mean data were used for statistical analysis.

### *In vitro* osteoclast differentiation and resorption

Three 8-week-old male TRPV1 knockout mice and three 8-week-old male C57BL/6 J mice were sacrificed, and the femurs were used to isolate the bone marrow-derived monocyte/macrophage cells (BMMs). The bone marrow cells were prepared using standard methods as previously described^41^. The BMMs were suspended in a 100 mm non-tissue culture plate with 10 ml medium (α-MEM containing 10% FBS and 1% pen-strep, Life Technologies, Beijing, China). The next day, the non-adherent cells were harvested and cultured with 10 ml medium supplemented with 30 ng/ml M-CSF (R&D Systems, Minneapolis, MN) for 3 days. After 3 days of culturing, the floating cells were removed, and the attached cells were harvested as osteoclast precursor cells. These cells were seeded at a density of 1 × 10^4^/well in a 96-well plate and cultured in culture medium supplemented with 30 ng/ml M-CSF and 50 ng/ml RANKL (R&D Systems, Minneapolis, MN). After 2 and 4 days of culture, the cells were harvested for real-time PCR analysis. After 4–5 days of culture, the cells were fixed with 10% formalin and analyzed with a tartrate-resistant acid phosphatase (TRAP) staining kit (Sigma-Aldrich, St. Louis, MO, USA).

A bovine bone slice was placed in a 96-well plate, and the osteoclast precursors were seeded on the surface of bone slice at a density of 10^4^ per well and cultured in culture medium supplemented with 30 ng/ml M-CSF and 50 ng/ml RANKL (R&D Systems, Minneapolis, MN). After 7 days, the cells were removed, and the slices were stained with hematoxylin. The resorbed area was quantified as described^42^.

### Polymerase chain reaction

DNA was extracted from the femurs of both groups. The primers for genotyping followed Jackson Laboratory recommendations. Primer oIMR1561 was used for wild-type forward 5′-TGGCTCATATTT GCCTTCAG-3′; primer 19923 was used for common reverse 5′-CAGCCCTAGGAGTTGATGGA-3′; and primer oIMR1627 was used for mutant forward 5′-TAAAGCGCATGCTCCAGACT-3′. The PCR program was as follows: 94 °C for 2 min; 35 cycles of 94 °C for 20 s, 58 °C for 15 s, 72 °C for 10 s; 72 °C for 5 min; and then held at 10 °C. The wild-type PCR product size was 289 bp, and the mutant PCR product size was 176 bp.

### Immunofluorescence

The sections of mouse femurs were rehydrated in an alcohol gradient and rinsed in distilled water. Antigen retrieval was conducted by microwave methods in sodium citrate solution. The sections were blocked, and TRPV1 primary antibody (Santa Cruz Biotechnology, USA) was incubated overnight at 4 °C. After being washed three times by PBS, the fluorescent secondary antibody was incubated, and DAPI was used to detect the nuclei. Pictures were captured by immunofluorescence microscopy. The relative immunofluorescence intensity was analyzed by Image J software as previous study^43^ and the relative TRPV1 positive osteoclasts were calculated as TRPV1 positive osteoclasts/bone marrow surface area by the Bioquant Osteo system.

### Calcium imaging

This method was performed according to previous methods^42^. Briefly, cultured osteoclasts stimulated by Rankl for 2 days were washed with standard working solution and incubated at 37 °C for 15 min in a 1 μM fura-2-AM solution (Molecular Probes). The cells were then imaged under an Olympus IX71 fluorescence microscope with a 20× objective. The emission intensities under 340 and 380 nm wavelengths were recorded every 1 s. The ratio of the emission densities (F340/F380) reflected the intracellular calcium concentration. The standard working solution contained the following (in mM): 135 NaCl, 4.0 KCl, 1.0 CaCl2, 1.0 MgCl2, 1.2 NaH2PO4, 10 glucose, and 10 HEPES. The pH was adjusted to 7.35 with NaOH.

### Osteogenic ability of bone marrow mesenchymal stromal cells

Primary bone marrow mesenchymal stromal cells were isolated from TRPV1 KO mice and WT mice. Briefly, the bone marrow cells were flushed out with syringes and resuspended in culture medium. The bone marrow cells were seeded in 100 mm dishes for 24 h. Then, the non-adherent cells were removed by rinsing with PBS. The attached cells were the bone marrow stromal cells and were seeded in 6-well plates at a density of 2 × 10^5^ per well and cultured for 7 days or 21 days in osteogenic medium. Then, the cells were harvested to assess the osteogenic-related genes and protein expression by real-time PCR and western blot analysis. Alizarin red staining was used to assess the calcium nodule-formation ability.

### Reverse transcription PCR and real-time PCR

Total RNA was isolated from differentiated bone marrow cells with TRIzol^®^ reagent (Invitrogen Life Technologies, Grand Island, NY, USA). Reverse-transcription PCR and real-time PCR were performed as previously described^43^. Actin mRNA expression was used as an internal control. The primers for actin and osteoclast-related gene mRNAs were as follows: dendritic cell-specific transmembrane protein (DC STAMP) forward/reverse 5′-CGAAGCTCCTTGAGAAACGA-3′/5′-GGACT- GGAAACCAGAAATGAA-3′, cathepsin K forward/reverse 5′-CGAAAAGAGCCTAGCGAACA-3′/5′-TGGGTA- GCAGCAGAAACTTG-3′, Atp6v0d2 forward/reverse 5′-AAGCCTTTGTTTGACGCTGT-3′/5′-GCCAGCACATTCATCTGTACC-3′.

Runx2 forward/reverse 5′-ATGCTTCATTCGCCTCACAAA-3′/5′-GCACTCACTGACTCGGTTGG-3′, ALP forward/reverse 5′-CCAACTCTTTTGTGCCAGAGA-3′/5′-GGCTACATTGGTGTTGAGCTTTT-3′, COL-1a forward/reverse 5′-GCTCCTCTTAGGGGCCACT-3′/5′-CCACGTCTCACCATTGGGG-3′, RANKL forward/reverse 5′-CGCTCTGTTCCTGTACTTTCG-3′/5′-GAGTCCTGCAAATCTGCGTT-3′, RANK forward/reverse 5′-CCAGGAGAGGCATTATGAGCA-3′/5′-ACTGTCGGAGGTAGGAGTGC-3′, OPG forward/reverse 5′- GGGCGTTACCTGGAGATCG-3′/5′-CGTTGTCATGTGTTGCATTTCC-3′.

### Western blotting

Total proteins were extracted by RIPA-lysis buffer (Beijing Solarbio Science & Technology, China), and nuclear proteins were extracted by a nuclear protein extraction kit (Applygen Technologies, China). The proteins were separated and transferred to a polyvinylidene fluoride membrane. The membrane was blocked using non-fat milk for 1 h at room temperature (RT). After incubation with primary antibody in TBS-T overnight at 4 °C, the membrane was washed extensively with TBS-T and then incubated with secondary antibody for 1 h at RT. The primary antibodies used in this study were β-actin, Runx2, NFATc1 (Santa Cruz Biotechnology, USA), and Lamin B (Bioworld Technology, USA).

### Statistical analysis

Statistical analyses were performed using GraphPad Prism (version 5.0) software. All data are expressed as the means ± SEMs. The Kolmogorov–Smirnov test was chosen to test the normal distribution. Student’s t-test was performed to compare differences in the cell differentiation and calcium signaling studies. One-way analysis of variance followed by Tukey’s post hoc analysis was performed to examine differences in the gene expression, histology, and microCT studies. p < 0.05 was considered significant.

## Additional Information

**How to cite this article**: He, LH. *et al*. TRPV1 deletion impaired fracture healing and inhibited osteoclast and osteoblast differentiation. *Sci. Rep.* **7**, 42385; doi: 10.1038/srep42385 (2017).

**Publisher's note:** Springer Nature remains neutral with regard to jurisdictional claims in published maps and institutional affiliations.

## Supplementary Material

Supplemental Information
