# Supplementary material for: TRPV1 deletion impaired fracture healing and inhibited osteoclast and osteoblast differentiation
Source: Sci Rep. 2017 Feb 22;7:42385. doi: 10.1038/srep42385 (PMC5320507; doi:10.1038/srep42385)
Supplement: Supplemental Information [file srep42385-s1.pdf]

TRPV1 deletion impaired fracture healing and inhibited osteoclast and osteoblast differentiation

Lin-Hai He[1¶], Meng Liu[1¶], Yang He[1], E Xiao[1], Lu Zhao[1], Ting Zhang[2], Hua-Qian Yang[3], Yi Zhang[1]*

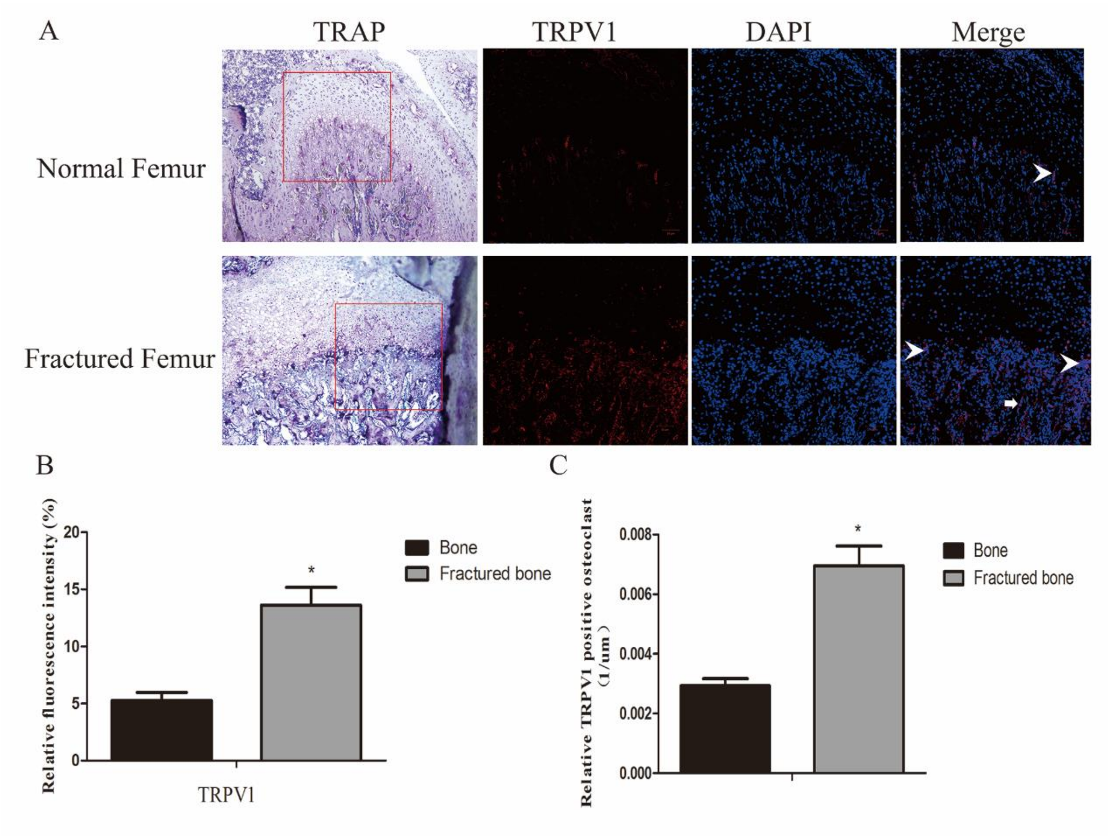

Supplemental figure 1 legend

TRPV1 expressions in bone and fracture healing. A: Left panel showed the TRAP staining. The rose color cells were TRAP-positive osteoclast. The right panel showed the TRPV1 expression in bone and fractured bone by immunofluorescence. The white arrow heads shows the TRPV1 positive osteoclast. The white arrow shows the bone lining cells. B: the quantification results of TRPV1 immunofluorescence intensity analyzed

by Image J software. C: the quantification results of TRPV1 positive osteoclasts analyzed by BIOQUANT OSTEO Bone Biology Research System.

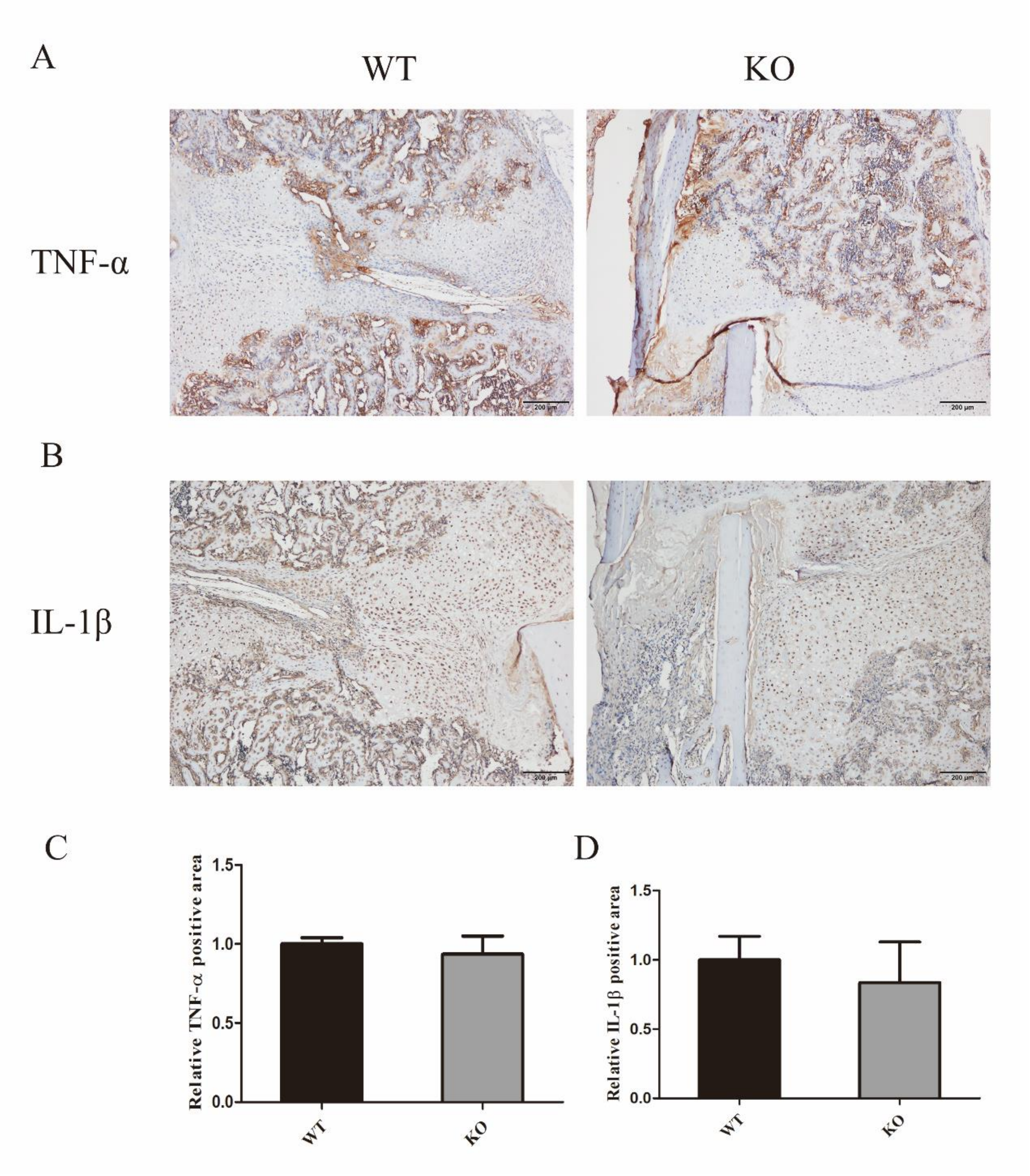

Supplemental figure 2 legend

Inflammation factors expression during fracture healing in WT and KO group. A: TNF-α expression in fracture callus detected by

immunohistochemistry staining. B: IL-1β expression in fracture callus detected by immunohistochemistry staining. C and D: quantification results of relative TNF-α and IL-1β positive area. No statistical differences of TNF-α and IL-1β expression between WT and KO group. *= $p < 0.05$
